# A Case of Erectile Tumor of the Orbit, Etc.

**Published:** 1853-12

**Authors:** Daniel Brainard

**Affiliations:** Professor of Surgery in Rush Medical College, Chicago, Illinois, late Vice-President of the American Medical Association, Etc.


### SELECTIONS.

From the London Lancet.

*A Case of Erectile Tumor of the Orbit, Cured by Infiltration with the Solution of the Lactate of Iron and Puncture with Hot Needles, after the Ligature of the Carotid Artery had failed;* with observations on the effect of that Solution in obliterating the Bloodveesels. By DANIEL BRAINARD, M. D., Professor of Surgery in Rush Medical College, Chicago, Illinois, late Vice-President of the American Medical Association, Etc.

WILLIAM W——, a farmer, aged thirty-four years, of good constitution, consulted me, August 1st, 1851, on account of a tumor of the left orbit. The eye was prominent, but could be closed; it rose perceptibly with the pulsation of the arteries, and gave a thrill on being touched. On placing the ear in contact with the tumor, a loud blowing sound was heard, which could be perceived, but less distinctly over the whole head. The veins of the face were prominent; the head was hot, and the arteries of the neck and head pulsated with unnatural force. Severe pain was often felt in the head, attended with nausea and vomiting; pressure on the left carotid instantly stopped the pulsation and sound.

*History.*—On the 14th of July, 1851, the patient received a severe kick from a horse on the left side of the lower jaw, which fractured it on the right side, and otherwise severely injured him. On recovering from the shock, he first perceived the sound in his head. This circumstance was calculated to mislead the surgeon into the opinion of a traumatic aneurism; but on a careful inquiry I found that the left eye had long differed from the other, principally in being short sighted. Finding compression could not be borne on account of the great tenderness, I advised the ligature of the left common carotid artery; but the patient's mind being unprepared for so serious an operation, he returned home, with the direction to adhere to a rigid diet, rest, and the application of ice or iced-water. Instead of strictly following this advice, he consulted various practitioners, regular and irregular, using by their direction every kind of application. About the first of November following he returned to me. The disease was greatly aggravated. The globe of the eye was protruded, the lids could not be closed, the conjunctiva was ulcerated, and the thrill could be clearly heard over the whole head. His general health had suffered much, and he has had several attacks, which he called bilious, and for which he had been purged and kept upon a strict diet for many days. Vomiting had increased the size of the tumor; he had severe pain in his head, and could only get relief from large doses of morphine. He had great heat in the head, and only slept by having cloths dipped in cold water kept constantly on it. He was now anxious to have the operation performed, which was done on the 11th November in the usual manner, the artery being tied about an inch below the division. No unusual symptoms occurred. Immediately after the operation the pulsation and sound in the tumor ceased.

The next day, the 12th, he complained of some pain in the right side of the head, and the capillaries of that side became greatly injected from the effort to establish the collateral circulation. A blood-letting of eight ounces gave great relief. The ligature came away on the fourteenth day. Immediately after the operation compression was attempted upon the tumor; but so acute was the sensibility that nothing more than the weight of a fold of linen, could be borne. Bags of pounded ice and salt were applied round it, and evaporating lotions upon it. Under this treatment, which, with a rigid diet, was continued, the tumor diminished in size; still on the third day after tying the artery a careful examination detected a slight pulsation and sound in the tumor. This continued, the tumor still subsiding. He left me on the 10th December. His health continued poor, and he was not able to attend to his business for several months. He noticed the sound of the circulation occasionally after the operation, and it gradually became more constant and stronger. He was often seized with what he called a bilious attack, which continued a day and a half, and was attended with severe retching and vomiting; at such times there was a marked increase of pain and swelling, which thereafter became constant. In October, 1852, the cornea had disappeared, the conjunctiva projected in a fungous shape, and several hemorrhages occurred, from rupture probably of the superficial vessels.

On the 11th November, 1852, just one year from the first operation, he returned to me. At this time the entire orbit was filled, the lower lid was concealed by the fungous projection, the eye pressed outwards and downwards, and at the root of the nose and inner part of the superciliary ridge was an elastic swelling, which had caused the absorption of the bone. It was at that point that the pulsations were the strongest, and the thrill the most distinct. The small vessels of the forehead and side of the nose were greatly enlarged, pulsated strongly, and the latter gave the peculiar thrill of the disease very sensibly. His general health was much impaired, and he had been able to come to the city by railroad only with great difficulty.

The question of the proper treatment to be employed in a case of erectile tumor of the orbit, when the ligature of one carotid has failed to effect a cure, is one of some difficulty. The attention is naturally turned to the other carotid; but even if the ligature of that were likely to succeed, the risk in this case was too great, since it was found that the compression of it for a few seconds rendered him perfectly insensible. None of the means resorted to for the purpose of obliterating this tissue had ever been tried on deeply-seated tumors like those of the orbit. On carefully reviewing them all, it was determined to try puncture with hot needles. This was done first on Nov. 13th, 1852; the needle used was of the size of a common knitting-needle, sharpened to a triangular point, and set in a handle of bone. After being heated in the flame of an alcohol lamp, it was plunged into the tumor about an inch from the root of the nose, in the course of the superciliary ridge, and carried downward and backward to a depth of over three inches; on withdrawing it, some bleeding followed, which was readily stopped by slight pressure. For two days there was little pain or swelling; but on the third day the inflammation was acute, the swelling extending over the face, and presenting an erysipelatous appearance. On the fifth day this began to decline, and in a week was nearly gone. While the inflammation was at its height the tumor was more firm and the thrill less distinct, but as it subsided the elasticity and pulsation rapidly returned.

Nov. 25th.—I repeated the operation, making the puncture half an inch nearer the nose, and carrying it to the depth of only one inch. The effects corresponded with those before described, but were a little less violent. On the third day the sound was diminished, and the firmness of the tumor very sensibly increased. On a careful examination it was found that the morbid tissue extended over the bridge of the nose and down to the inner canthus of the right eye, where a thrill could be distinctly felt.

Dec. 2nd.—A puncture was made on the left side of the bridge of the nose, the needle beeing carried obliqely upward and to the left side. The inflammation resulting from this puncture was acute, and a superficial supuration occurred about it. The thrill upon the right side of the nose quite disappeared.

The effect of these three punctures was to limit the spread of the tissue upon the forehead and nose; but the inflammation although acute and extensive, was superficial, and the mass of the disease at the centre was still unaffected. It was evident that the needles cooled in passing through the tissues, so as not to cauterize much below the surface.

It was therefore determined to change the treatment, and inject a fluid into the centre of the tumor capable of effecting its obliteration. For this purpose a solution of the lactate of iron, of eight grains to one fluid ounce of distilled water, filtered through paper, was preferred. The reasons for believing in the safety of this remedy will be given below.

14th.—I punctured the tumor at its most prominent part with the infiltrating canula, carrying it to the depth of about an inch; on withdrawing the stylet arterial blood followed. A fluid drachm of the above-named solution was immediately thrown in with a small syringe, constructed for the purpose and the canula withdrawn. The immediate effect was an intense pain in the left temporal region and a flushing of the face, which latter only lasted a few seconds. A chill followed, accompanied with nausea and vomiting. Reaction took place in an hour, but the vomiting continued, and for twenty-four hours all drinks were ejected; pulse sixty-three.

15th.—Vomiting continues; pain less; upper lid much swollen; pulse 65.

16th.—Vomiting less; no pain; has slept well; pulse 60; swelling increased, and so tender as not to bear the slighest touch.

23rd.—For the last six days the vomiting has gradually diminished; the pulse is natural; the tumor is less tender, firm, and the pulsation perceived only at the external angle; frequent lancinating pain is felt in the orbit.

During the whole of this treatment, both of puncture and infiltration, the head has been kept enveloped in bladders filled with a freezing mixture of pounded ice and salt; which was very grateful to the patient. He now began to complain of its being too cold. The heat in the head was reduced to the natural standard, and from the time of the infiltrati on neither thrill or sound has been perceived. The veins of the face were much diminished in size, and the pulsation of the arteries reduced to its natural state. A slight pulsation was still perceptible at the angle of the eye, a puncture was made at that point, with a hot needle, on Jan, 4th, 1853.

Jan. 10th.—From the time of the last puncture no pulsation has been perceived, the swelling subsiding. At this time an opening was found to exist on the anterior surface of the globe of the eye, which still remains protruded between the lids. This was followed by severe inflammation of the globe, which lasted several days. The discharge from the opening was at first the humours of the eye, afterwards pus, but no blood.

Feb. 5th.—Swelling gradually subsiding; tumor firm, no pulsation; but little pain. The patient slept for the first time for more than a year without some one to keep wet cloths upon his eye, dressed himself, and walked about the house; health good.

March 5th.—Swelling entirely disappeared; the globe of the eye perfectly collapsed; lids closed.

June 6th.—The patient has been pursuing his ordinary occupation for three months; his health appears perfectly restored.—The left orbit seems entirely excavated and free from all disease.

At this time he was presented at the annual meeting of the Illinois State Medical Society, and examined by a large number of its members, who concurred in the opinion that the cure might be regarded as perfect.

*Remarks.*—It will readily be understood that so serious an operation as throwing a fluid drachm of a solution of the lactate of iron, at a point almost to saturation, directly into the current of the dirculation, would not be ventured upon without the most satisfactory reasons for regarding it, firstly, as safe; secondly, as effectual. I propose now to offer briefly some of the reasons for so regarding it.

In 1850, I was engaged in making inquiries concerning the effects of this salt in certain diseased states of the system, and was induced, from certain theoretical views, to try the effect when thrown into the veins. Before doing this upon the human subject, I tried it upon a dog, dissolving, rather imperfectly, ten grs. of it in one fluid ounce of distilled water, This was thrown at once into the saphena vein of a middle-sized dog, and the experiment was twice repeated without any ill effect.

In December, 1851, I first used it upon the human subject, using in the course of eight weeks nineteen grains of the salt in solution. The largest amount injected at one time was three grs., dissolved in three drachms of distilled water. The number of times it was employed in this subject was nine. It had no ill effects, but the contrary; what is pertinent to our present subject is, that each of the veins into which it was thrown at the bend of the arm was found after a certain length of time to be obliterated, and converted into a firm cord, without there being produced either pain or perceptible inflammation. I have since used it in four other cases,—in two of them once, and in the other two twice each,—without any unfavorable effects. In one of these cases, in which it was used twice, a vein was opened a second time, three days after the first operation, but it was found filled at that point with coagulum, which of course was not disturbed, but the injection made by another vein. Its tendency to obliterate the veins by a gradual process of thickening their coats and obstructing the circulation, was thus made sufficiently apparent, while its safety, used with requisite precautions, was clearly demonstrated. As these cases related solely to the veins, before using it in the case of tumor of the orbit, in which it might enter directly into the arterial circulation, it was thought proper to try it upon an artery in a dog. Accordingly, three grains of the lactate dissolved in three fluid drachms of distilled water, were thrown into the carotid artery of a small dog, and the vessel tied. The only effect was a slight irregularity of the action of the heart, which continued but a few seconds. As this entered directly into the cerebral circulation, its safety when used in much smaller quantity, in the human subject, might legitimately be inferred.—The result, as used upon the tumor, fully justified its employment, although the effects produced were somewhat severe. They were greatly less so, and less dangerous, than those resulting from the ligature of the carotid artery; and used in smaller quantity, repeated if necessary, it seems to promise relief without the necessity of resorting to that operation.

I may add here, that I have since used the same solution upon an erectile tumor of a venous character, of the size of a pigeon's egg, upon the inside of the lower lip, by injecting it at three different times, without any other effect than a slight tenderness, followed by gradual diminution of size and induration of tissue; but the cure is not yet complete.

The applicability of this practice to the treatment of varicose veins and aneurisms will readily suggest itself, but further experiments will be required to determine to what extent it can be relied upon in these cases. The number of the *Revue Medico Chirurgicale de Paris* for May, 1853, contains some interesting experiments by M. Pravaz, M. Giraldes, and M. Debout, on the coagulation of the blood in the arteries of animals by the injection of a solution of the perchloride of iron, and also three cases in which it had been used for the treatment of vascular tumor in the human subject, in each of which suppuration, and in one, gangrene followed. These results are not of a nature to encourage further trials of that substance; but if we take into consideration that the solution of the perchloride of iron is a substance foreign to the normal constitution of the blood, and produces instant coagulation when brought in contact with it, whereas the solution of the lacate of iron is composed of elements which enter into the composition of the blood; that when thrown into the living vessels it does not coagulate it, but produces a thickening of the coats and a deposition of coagulable lymph from subacute inflammation; the difference between the two results will be readily understood. It is probable that the solution of the lactate, when thrown into the vessels immediately undergoes decomposition, the acid combining with the soda of the blood, and the base passing into a higher state of oxydation in which it naturally exists in that fluid. In no case has this solution, when thrown into the blood or into a vascular tumor, shown a tendency to produce suppuration, but a slow inflammation of an adhesive kind and limited extent. In one case, where it was inadvertently pressed in small quantity into the subcutaneous cellular tissue, instead of into the vein as intended, a hard swelling having all the appearance of a boil or small carbuncle was the result; but even this effect will not be produced by it, when used in that manner, unless the solution be in a certain degree concentrated.

